# Genetic and serologic surveillance of canine (CIV) and equine (EIV) influenza virus in Nuevo León State, México

**DOI:** 10.7717/peerj.8239

**Published:** 2019-12-17

**Authors:** Claudia B. Plata-Hipólito, Sibilina Cedillo-Rosales, Nelson Obregón-Macías, Carlos E. Hernández-Luna, Cristina Rodríguez-Padilla, Reyes S. Tamez-Guerra, Juan F. Contreras-Cordero

**Affiliations:** 1 Universidad Autónoma de Nuevo León, Facultad de Ciencias Biológicas, Laboratorio de Inmunología y Virología, San Nicolás de los Garza, Nuevo León, México; 2 Universidad Autónoma de Nuevo León, Facultad de Medicina Veterinaria y Zootecnia, Departamento de Virología, Escobedo, Nuevo León, México; 3 Universidad Autónoma de Nuevo León, Facultad de Medicina Veterinaria y Zootecnia, Departamento de Grandes Especies, Escobedo, Nuevo León, México; 4 Universidad Autónoma de Nuevo León, Facultad de Ciencias Biológicas, Departamento de Química, San Nicolás de los Garza, Nuevo León, México

**Keywords:** Equine Influenza Virus (EIV), Canine Influenza Virus (CIV), Matrix gene (M), Polyclonal antibodies

## Abstract

**Background:**

Despite the uncontrolled distribution of the Influenza A virus through wild birds, the detection of canine influenza virus and equine influenza virus in Mexico was absent until now. Recently, outbreaks of equine and canine influenza have been reported around the world; the virus spreads quickly among animals and there is potential for zoonotic transmission.

**Methods:**

Amplification of the Influenza A virus matrix gene from necropsies, nasal and conjunctival swabs from trash service horses and pets/stray dogs was performed through RT-PCR. The seroprevalence was carried out through Sandwich enzyme-linked immunosorbent assay system using the M1 recombinant protein and polyclonal antibodies anti-M1.

**Results:**

The matrix gene was amplified from 13 (19.11%) nasal swabs, two (2.94%) conjunctival swabs and five (7.35%) lung necropsies, giving a total of 20 (29.41%) positive samples in a pet dog population. A total of six (75%) positive samples of equine nasal swab were amplified. Sequence analysis showed 96–99% identity with sequences of Influenza A virus matrix gene present in H1N1, H1N2 and H3N2 subtypes. The phylogenetic analysis of the sequences revealed higher identity with matrix gene sequences detected from zoonotic isolates of subtype H1N1/2009. The detection of anti-M1 antibodies in stray dogs showed a prevalence of 123 (100%) of the sampled population, whereas in horses, 114 (92.68%) positivity was obtained.

**Conclusion:**

The results unveil the prevalence of Influenza A virus in the population of horses and dogs in the state of Nuevo Leon, which could indicate a possible outbreak of equine and Canine Influenza in Mexico. We suggest that the prevalence of Influenza virus in companion animals be monitored to investigate its epizootic and zoonotic potential, in addition to encouraging the regulation of vaccination in these animal species in order to improve their quality of life.

## Introduction

Influenza is a highly contagious disease caused by Influenza virus. In nature, this virus has a wide range of hosts and is responsible for panzootic, epizootics and pandemics around the world, affecting the health of both humans and animals, and inflicting a heavy socioeconomic burden.

Usually, most viruses are restricted to their original host, however, there is evidence of the emergence of zoonotic strains that can replicate and spread within and between animal species (Wiwanitkit, 2014).

Canine Influenza Virus (CIV) is a zoonotic infection that spread from horses to dogs (Crawford et al., 2005) and birds (Song et al., 2008). The virus has since established within the population of pet and stray dogs, occasionally causing epizootic outbreaks in overcrowded sites such as animal shelters. At present, CIV is currently distributed in at least 30 states of the United States (Voorhees et al., 2017; Centers for Disease Control and Prevention, 2019). The detection of Equine Influenza Virus (EIV) occurs in sporadic outbreaks around the world, where essentially, the disease is distributed among unvaccinated horses in precarious animal health conditions (Silva et al., 2014; Singh et al., 2018; Sack et al., 2019).

Several factors could contribute to the emergence of the Influenza A virus in companion animals such as the absence of vaccination (Silva et al., 2014), co-infection with other respiratory agents (Metzger & Sun, 2013) and co-existence with infected animals in poor health conditions (Holt, Mover & Brown, 2010; Hayward et al., 2010). For that reason, monitoring the presence of the Influenza virus in these animal populations is necessary to establish preventive measures against this virus.

Influenza virus is a member of the *Orthomyxoviridae* family. This family comprises four species: Influenza A, B, C and D virus, all of them identified through antigenic differences on the nucleoprotein and matrix protein (M) (Wright et al., 1995). Because of its high conservation within the viral genome (Furuse et al., 2009; Chander et al., 2013), several studies use the matrix gene for the detection of Influenza A virus in diverse animal species (Wallace et al., 1999; Herrmann, Larsson & Zweygberg, 2001; Widjaja et al., 2004; Harmon et al., 2010).

In Mexico, the presence of the virus in canine and equine populations has been suspected due to the detection of antibodies in pet dogs (Ramírez-Martínez et al., 2013) and horses (Blitvich et al., 2010), however, the virus itself has not been detected.

Mexico has a population of more than six million horses intended for different activities. Particularly, waste transportation is characterized by poor working conditions and constant contact with other animal species susceptible to Influenza A virus (Instituto Nacional de Estadística y Geografía, 2014). The stray dog population exceeds 18 million (Cortez-Aguirre et al., 2018), and the probability of Influenza A virus spreading among these animals is high (Gencay et al., 2004; Kasempimolporn et al., 2007; Levy et al., 2008; Beeleer, 2009) because of the absence of a vaccine against CIV in Mexico. The aim of this study was to determine the presence of Influenza A virus in a populations of trash service horses and pet/stray dogs in Nuevo Leon, Mexico through the detection of the matrix gene as well as the prevalence of the virus in this animal population and its need for vaccine protection.

## Materials and Methods

### Ethics statement

All animal experiments were approved by the Animal Research and Welfare Ethics Committee (CEIBA-2018-024) of the Laboratory of Immunology and Virology of the College of Biological Sciences (FCB), Universidad Autónoma de Nuevo León (UANL) and the sampling was made under the indications of the NOM-062-ZOO-1999. Informed consent was obtained from the owners of the animals for the collection of additional information.

### Study area and collection of samples

The samples were obtained in the period between March of 2013 and December of 2015 from nine municipalities (Apodaca, Cadereyta Jimenez, Escobedo, Guadalupe, Juarez, Montemorelos, Monterrey, San Nicolas de los Garza and Zuazua) of Nuevo Leon, Mexico. The College of Veterinary Medicine and Zootechnics (FMVZ), UANL sampled domestic dogs with acute respiratory symptoms. Of this dog population, 58 nasal swabs, five lung necropsies and five conjunctival swabs were obtained. For the canine serological study, 123 samples were collected from stray dogs in Guadalupe and Juarez, Nuevo Leon. None of the dogs had a travel history and none had been vaccinated against CIV. Samples (123 sera and eight nasal swabs) were also collected from trash service horses. Swabs and necropsies were kept in Minimum Essential Medium additioned with antibiotic–antimycotic Gibco^®^ (Thermo Fisher Scientific, Waltham, MA, USA) 2% and stored at −70 °C until use. Blood samples were obtained from venipuncture and centrifuged at 3,000 rpm to obtain the serum, which was stored at −20 °C until use.

### Sample collection

Samples of necropsies and nasal and conjunctival swabs were used to obtain viral RNA. The sampling frame was the presence of respiratory symptoms characteristic of CIV and EIV in the populations analyzed. Analysis of seroprevalence in stray dogs and trash service horses was performed on randomly selected samples.

### RNA isolation and M gene amplification

Viral RNA was purified from lung necropsies, nasal and conjunctival swabs using AxyPrep™ Body Fluid Viral DNA/RNA Miniprep Kit (AxygenBioscience, Union City, CA, USA) following the manufacturer’s protocol. Coding region of 756 bp of M gene was obtained by SuperScript^®^ III One-Step RT-PCR system with Platinum^®^ Taq High Fidelity (Thermo Fisher Scientific, Waltham, MA, USA) using M specific primers (5′-CACGGATCCAAGATGAGTCTTCTAACCGAC-3′ and 5′-CACGTCGACAGGATCACTTGAATCGCTGCA-3′). Subsequently, a second PCR was performed using oligonucleotides recommended by the World Health Organization (2009) for Influenza A virus detection (5′-ATGAGYCTTYTAACCGAGGTCGAAACG-3′ and 5′-TGGACAAANCGTCTACGCTGCAG-3′) obtaining a 244 bp PCR product. RNA from the vaccines Fluvac Innovator Pfizer Equine Influenza Vaccine (A2/Kentucky/97) and Vanguard Plus 5 L4 Vaccine were used as controls. Amplification product was visualized on a 1.5% agarose gel and then sequenced to confirm its genetic identity.

### Sequence analysis and phylogenetic tree

Products of the expected size (244 bp), were purified using Wizard^®^ SV Gel and PCR clean-up system (Promega, Madison, WI, USA) and directly sequenced by an automated system (Perkin Elmer/Applied Biosystem, Foster City, CA, USA) at the Institute of Biotechnology of the Universidad Nacional Autónoma de México (IBT-UNAM). The sequence analysis was done using CLUSTAL W/BioEdit sequence alignment v7.0 (Hall, 1999) and MEGAX software (Kumar et al., 2018). The partial sequences of M1 obtained in this study were submitted to the GenBank database under accession numbers MH463596, MH463597, MH463598, MH463599, MH463600, MH463601 and MH463602. The phylogenetic tree contains sequences corresponding to the M1 matrix gene of Influenza A virus obtained from the Influenza Virus Resource (NCBI) database. Zoonotic reassortants: MK690088, CY050848, MG254077, GU471689; Contemporary isolates: AY737298, HQ237603, MF173290, MH363692, MK690096 and Ancestral strains of CIV and EIV: CY109269, GU433346, DQ124160, CY067361, DQ222916, CY028837, Outgroup: CY125948 and KR077935. Maximum likelihood Jones–Taylor–Thornton model was performed to the construction of the phylogenetic tree.

### M1 recombinant matrix protein

A feces sample of a wild bird positive for Influenza A virus (kindly provided by Dr. Jose Ignacio Gonzalez Rojas of the Laboratory of Ornithology, College of Biological Sciences, UANL) was processed to obtain the coding sequence of the M1 gene. The sequence whose identity coincided 100% with the matrix gene present in subtype H1N1/2009 was subcloned into the expression vector pET-28a (+) (Promega, Madison, WI, USA). Protein expression was carried out in competent *Escherichia coli* BL21 bacteria with pET-28 (+) and IPTG expression system. Bacteria with 0.4 OD (600 nm) of biomass without IPTG was taken before induction as negative control. Subsequently, the expression was induced and after 5 h of incubation the protein was purified with an OD of 1.6. Bacteria lysis was carried out by lysozymes and sonication. Purification by HisTrap™ HP columns (GE Healthcare, Chicago, IL, USA) under denaturing conditions with urea (4 M) was performed. The quantification of the purified protein fractions was performed by a semi quantitative–qualitative method comparing band intensity in the SDS-Page, using bovine albumin fractions as control. The purified protein was analyzed by Western blot to confirm its identity using a monoclonal antibody against influenza virus M1 protein (Abcam, Cambridge, UK).

### Polyclonal antibodies

New Zealand White rabbits were immunized subcutaneously with recombinant matrix protein at six different points. For the primary immunization, 1 mg of matrix protein was administered with Freund’s complete adjuvant. For booster immunizations, 0.5 mg of protein was administered with Freund’s incomplete adjuvant 15 days after the primary immunization and once more 2 weeks after that (Leenaars & Hendriksen, 2005). Blood was collected 15 days after the final immunization and sera was obtained. The conditions of animal protection were followed by NOM-062-ZOO-1999.

### Sandwich ELISA assay

Microtiter plates were coated with dog/horse antibodies diluted in carbonate–bicarbonate buffer pH 9.6 and incubated overnight at 4 °C. After two washes with phosphate-buffered saline (PBS), the plates were blocked with 5% nonfat dry milk in PBS at 37 °C for 1 h. After two washes, recombinant matrix protein was added at a concentration of 40 µg/mL and incubated at 37 °C for 1 h. The anti-M1 antibody (1:7,000) produced in rabbit was added and incubated at 37 °C for 1 h after two washes. Peroxidase-conjugated protein A (Amersham, Buckinghamshire, UK) was added and incubated at 37 °C for 1 h, after two washes. The plates were then washed two times and the ABTS (2,2′-azino-bis (3-ethylbenzothuazoline-6-sulphonic acid)) peroxidase substrate (KPL, Gaithersburg, MD, USA) was added and incubated at 37 °C for 30 min. The absorbance at 405 nm was measured using an automatic microplate reader (Digital and Analog Systems, Roma, Italy). Human serum positive for Influenza H1N1/2009 and fetal bovine serum were used as a positive and negative control, respectively. The cutoff value was defined as the mean of the negative control OD 405 nm values plus three standard deviations.

## Results

Canines and equines with acute respiratory disease were analyzed for the detection of CIV and EIV through the amplification of the matrix gene of the Influenza A virus. The collection of samples was carried out during the period between March of 2013 and December of 2015 in nine municipalities of Nuevo León State, Mexico (Table 1).

**Table 1:** Positive samples to Influenza A virus by matrix gene detection in canines and equines from Nuevo Leon State, Mexico.

Host	Type of sample	Municipalities	Number of samples	Number of positives (%)
Canines	Nasal swab	Monterrey Escobedo San Nicolas de los Garza Apodaca Guadalupe	4 13 32 6 3	2 (15.38) 6 (46.1) 3 (23) 1 (7.6) 1 (7.6)
	Conjunctival swab	San Nicolas de los Garza Apodaca	3 2	1 (20) 1 (20)
	Lung biopsy	ND	5	5 (100)
Equines	Nasal swab	Montemorelos	1	1 (16)
		Cadereyta	5	4 (50)
		Monterrey	1	0
		San Nicolas de los Garza	1	1 (16)
		Total	76	26 (34.2)

### Matrix gene amplification of Influenza A virus

Of the total samples, the matrix gene was amplified in 13 (19.11%) nasal swabs, two (2.94%) conjunctival swabs and five (7.35%) lung necropsies, giving a total of 20 (29.41%) positive samples in dog population. Meanwhile, six (75%) positive samples of equine nasal swab were amplified. All viruses detected were isolated from unvaccinated animals (Table 1).

### Sequence analysis of matrix gene

**Figure 1:**
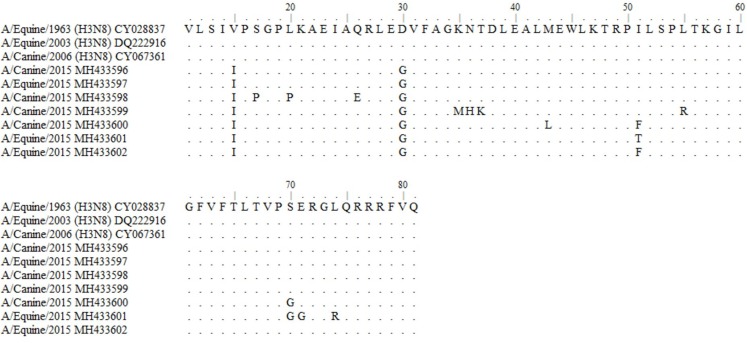
Alignment of reference amino acid sequences of American equine and canine lineage and deduced amino acid sequences obtained in this study. GenBank: A/equine/Miami/1/1963 (H3N8): CY028837; A/equine/Wisconsin/1/03 (H3N8): DQ222916; A/canine/Colorado/8880/2006(H3N8): CY067361.

Amplicons of 244 bp corresponding to the partial sequence of the matrix gene (M) from canines and equine samples were sequenced. Nucleotide alignments of the analyzed sequences showed a 96–99% identity with sequences of M1 gene of Influenza A virus. These isolates were compared with matrix sequences of representative strains of clade 1 from American lineage (Florida): A/eq/Miami/63, A/eq/Wisconsin/03 and A/canine/Colorado/06, presents 2–6 amino acid substitutions (97.18–99.15% identity). This comparison showed genetic variability in the coding region between amino acids 10 and 80 of M1, observing greater genetic variability in the canine sequences than in the equine sequences (Fig. 1).

**Figure 2:**
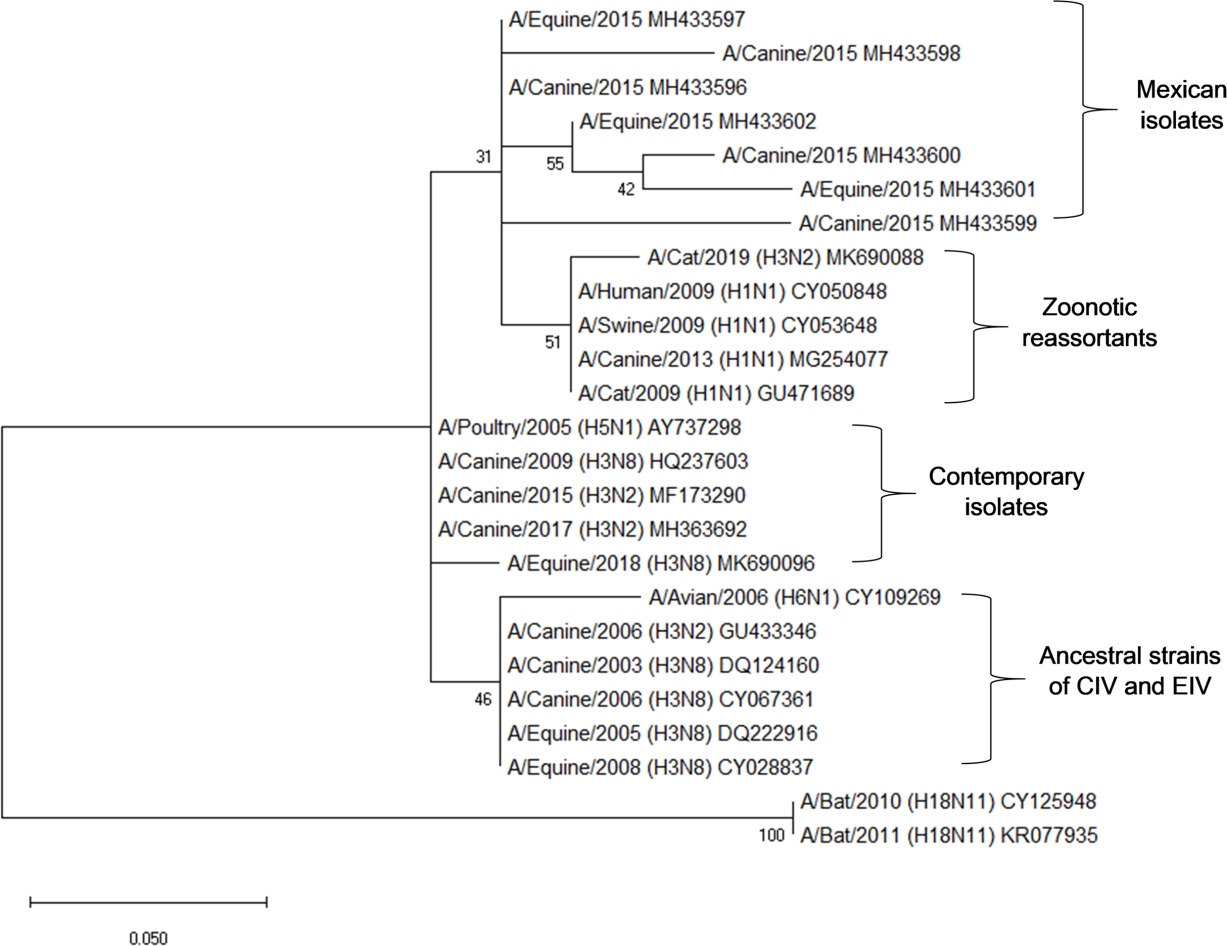
Phylogenetic analysis of deduced M1 protein amino acid sequences of canine and equine influenza virus isolations. The tree was constructed using the neighbor joining method; bootstrap of 1,000 replication (MEGAX).

The phylogenetic analysis of the sequences shows proximity with zoonotic and potentially pandemic strains such as the H3N2, H1N2 and H1N1/2009. Sequences of equine, canine and avian origin were included to determine the genetic divergence in the partial sequence of the M gene. In this analysis, it is evidenced how the sequences obtained in this study (Mexican isolates) possess a genetic identity of 95.78–97% with matrix sequences of H3N2 and H1N1 isolates obtained from domestic cats, as well as matrix sequences from isolates of the pandemic strain H1N1/2009 from swine and humans (Zoonotic reassortants). Mexican isolates also have an identity of 92.96–98.6% with avian, equine and canine contemporary strains. Partial sequences of the M gene from the ancestral strain of H3N2 and H3N8 of CIV and EIV were included from GenBank, obtaining an identity of 91.55–97.19% (Fig. 2).

### Seroprevalence and symptomatology

A total of 123 sera from canines and 123 sera from equines obtained from nine municipalities were tested by Sandwich enzyme-linked immunosorbent assay (ELISA) system. Polyclonal antibodies against recombinant Influenza A matrix protein were founded in the 123 (100%) sera in canines. At least 21.46% of canine population had been vaccinated against other respiratory viral agents. Meanwhile, 114 (92.68%) of equines presented polyclonal antibodies against Influenza virus. Vaccine information in equines was only reported against tetanus toxoid.

The population of canines resulting positive for CIV had at least three predominant symptoms: conjunctivitis, runny nose and sneezing. Conjunctivitis and runny nose were presented in 27.94% of the sampled population, whereas sneezing was observed in at least 10 dogs positive for CIV. The equines reported fewer symptoms, predominantly runny nose and sneezing, both in the sampled population and in the positive samples for EIV (Table 2).

**Table 2:** Predominant symptoms in the population of canines and equines sampled.

Symptoms	Number (%) of canines with reported symptoms	Number (%) of equines with reported symptoms	Positive samples	
			Canines	Equines
Anorexy	18 (26.47)	2 (25)	10	2
Cough	28 (41.17)	4 (50)	11	3
Respiratory difficulty	16 (23.52)	4 (50)	10	2
Fever	12 (17.64)	2 (25)	7	2
Conjunctivitis	39 (57.35)	Absent	19	Absent
Runny nose	34 (50)	8 (100)	13	6
Sneezing	20 (29.41)	7 (87.5)	11	3
Emesis	4 (5.88)	Absent	1	Absent

## Discussion

Although outbreaks of Influenza A virus in dogs and horse populations worldwide have been reported, CIV and EIV had not been detected in Mexico until now. However, the presence of the virus in pets had already been reported in other parts of the world increasing its incidence in domestic animals (Payungporn et al., 2008; Song et al., 2008; Lee et al., 2010; Lin et al., 2015). In this study, 58 nasal fluid, five lung necropsies and five conjunctival swabs samples from pet dogs with acute respiratory symptoms were used to detect Influenza A virus through matrix gene amplification with the WHO recommended primers. Of them 13 (19.11%) nasal fluids, five (7.35%) lung necropsies and two (2.94%) conjunctival swabs from pet dogs with were positive. Although this value is higher than reported in other studies, this could be explained because all of the sampled animals presented classic symptoms of Influenza.

All lung necropsies presented co-infection of Influenza A and other respiratory agents such as Canine Adenovirus type 2, Canine Distemper Virus and Canine Parainfluenza. These co-infections could be an important factor in the severe respiratory diseases suffered by the pet dogs (Unpublished). Co-infection could aggravate the disease and induce the early death of animals (Löhr et al., 2010; Kash et al., 2011; Metzger & Sun, 2013). The detection of viral matrix gene from conjunctival swab samples could reinforce that Influenza A virus using the eye as a portal of entry as well as a primary site of virus replication (Belser et al., 2018).

Six (75%) nasal fluid samples from trash service horses that presented characteristic symptoms of Influenza A virus were positive to matrix gene amplification. Although this percentage could be considered high, a recent study showed 96% of EIV detection in nasopharyngeal swabs from equines in Malaysia during an outbreak in 2015 (Toh et al., 2019).

Although there are many viruses that infect the respiratory tract and they could induce similar symptoms, at present, symptoms such as conjunctivitis, runny nose and sneezing has been observed as the main symptoms related to the detection of CIV and EIV (Spickler, 2016; Singh et al., 2018). These symptoms that were present in the population of canine and equine analyzed.

In Mexico, there is no CIV vaccination available as a preventive measure against the disease. The vaccination against EIV is not mandatory, thus, the immune status against EIV is unknown. However, the incidence of EIV in 23 states of United States during 2015 supports the possibility of an outbreak within the equine population (Sack et al., 2019). This population of animals are exposed to multiple risk factors such as work injuries, the contact with waste, and interaction with different animal species (Rimbaud et al., 2006).

The partial nucleotide sequences of the matrix gene showed 96.24–100% of homology with matrix gene sequences of H1N1, H1N2 and H3N2 subtypes from swine in North America obtained from GenBank. Several studies have shown an incidence of H1N1/2009 subtype in dogs (Dundon et al., 2010; Ozawa & Kawaoka, 2013; Su et al., 2014; Moon et al., 2015), as well as possible reassortments between H1N1 with H3N2 (Song et al., 2012; Na et al., 2015; Wang et al., 2017).

The comparison of amino acid deduced sequences from equine and canine matrix gene sequences from ancestral strains A/Equine/1963 (H3N8), A/Equine/2003 (H3N8) and A/Canine/2006 (H3N8) indicates that most the residues are highly conserved, obtaining 92.96–97.19% of consensus sequences. Divergences at positions V15I, L20P, D30G, N36H, T37K do not represent a significant change in the protein because they are synonymous amino acids to those present in the ancestral strains. Besides, the mutations at positions S17P, Q26E, K35M, S70G, E71G and L74R are present in the C-terminal domain which is characterized as high tolerant to mutations that do not compromise the functionality of the viral protein (Hom et al., 2019).

Interestingly, the phylogenetic analysis of the sequences reveals the phylogenetic closeness with M gene sequences present in strains of zoonotic outbreaks in cats, dogs, swine and humans: (A/Cat/USA/047732/18, A/cat/OR/29573/2009, A/Canine/Ggx/WZ1/2013, A/Sw/4/Mexico/2009, A/Hu/Mx/007/2009) with H1N1 and H3N2 subtype.

The study of Influenza A virus in dogs and horses in Mexico has been limited to the detection of antibodies against the virus. The high seroprevalence obtained in the analysis of antibodies from stray dogs and equines evidences the susceptibility to the Influenza A virus among animals in precarious conditions of care. It would be interesting to know whether these antibodies are present in protective levels.

Pet dogs’ studies in Mexico have shown a low seropositivity against H1N1/2009, H3N2 (0.9%) and H3N8 subtype (4%) (Ramírez-Martínez et al., 2013). On the other hand, studies of canines from animal shelters suggest that prolonged exposure to the virus from other infected dogs considerably increases the seropositivity of the animals analyzed from 15% to 71% after 8 days of confinement (Holt, Mover & Brown, 2010). A limiting factor in this study is the lack of information about the permanence of stray dogs in animal shelters.

The seropositivity of horses analyzed in this study (92.68%) was greater than 57.89% reported in Nuevo León in 2010 (Blitvich et al., 2010). However, this percentage is similar to that reported by Silva et al. (2014) in unvaccinated horses from Brazil during an outbreak in 2014, where they found a seroprevalence of 92%.

Interspecies transmission of Influenza A virus is still unclear. Nevertheless, some animal species could have a role as a mixing vessel of segments that would give rise to potentially zoonotic subtypes. Among these species, domestic animals such as pet dogs and horses stand out (Parrish, Murcia & Holmes, 2015; Chen et al., 2018). Because these species are companion animals for humans, the presence of Influenza virus in these species could represent a public health risk for both human and animal populations.

## Conclusions

Our results unveil the prevalence of Influenza A virus in the population of horses and dogs in the state of Nuevo León, Mexico. Because CIV and EIV have spread horizontally among the population of dogs and horses across the United States, the detection of the Influenza A virus in canines and horses in the state of Nuevo Leon, Mexico is not surprising.

The detection of the M gene and antibodies against the matrix protein provide an effective identification of the Influenza A virus in dogs and horses because of the genetic and antigenic stability of the M gene. This becomes important due to the constant variability of the virus and its need for constant epidemiological surveillance. Vaccination plays an essential role in stopping the spread of the virus, so we suggest that vaccination systems should be updated and regulated to improve the health conditions of species susceptible to the virus.

## Supplemental Information

10.7717/peerj.8239/supp-1 Supplemental Information 1 Supplemental Information Supplemental information of sampled collection used in this research.

## Additional Information and Declarations

### DNA Deposition

The following information was supplied regarding the deposition of DNA sequences: The partial sequences of M1 obtained in this study are available at GenBank: MH463596, MH463597, MH463598, MH463599, MH463600, MH463601 and MH463602.

### Data Availability

The following information was supplied regarding data availability: The raw data are available in the Supplemental Files.
